# A novel *Glycine soja* homeodomain-leucine zipper (HD-Zip) I gene, *Gshdz4*, positively regulates bicarbonate tolerance and responds to osmotic stress in *Arabidopsis*

**DOI:** 10.1186/s12870-016-0872-7

**Published:** 2016-08-24

**Authors:** Lei Cao, Yang Yu, Huizi DuanMu, Chao Chen, Xiangbo Duan, Pinghui Zhu, Ranran Chen, Qiang Li, Yanming Zhu, Xiaodong Ding

**Affiliations:** Key Laboratory of Agricultural Biological Functional Genes, Northeast Agricultural University, Harbin, 150030 China

**Keywords:** *Gshdz4*, Transcription factor, Bicarbonate tolerance, Osmotic stress, *Glycine soja*, *Arabidopsis*

## Abstract

**Background:**

Wild soybean (*Glycine soja*) is a highly adaptive plant species which can grow well in saline-alkaline soils. In soybean genome, there exist about 140 HD-Zip (Homeodomain-leucine Zipper) genes. HD-Zip transcription factor family is one of the largest plant specific superfamilies and plays important roles in response to abiotic stresses. Although HD-Zip transcription factors have been broadly reported to be involved in plant resistance to abiotic stresses like salt and drought, their roles in response to bicarbonate stress is largely unknown.

**Results:**

From our previous transcriptome profile analysis of wild soybean treated by 50 mM NaHCO_3_, we identified an HD-Zip gene (*Gshdz4*) which showed high response to the alkaline stress. Our result of qRT-PCR showed that the expression of *Gshdz4* was induced by alkaline stress (NaHCO_3_) in both leaves and roots of wild soybean. Overexpression of *Gshdz4* in *Arabidopsis* resulted in enhanced tolerance to NaHCO_3_ and KHCO_3_ during the process of plant growth and development. However, the growths of transgenic and WT plants were not significantly different on the medium with high pH adjusted by KOH, implicating *Gshdz4* is only responsible for resisting HCO_3_^−^ but not high pH. The transgenic plants had less MDA contents but higher POD activities and chlorophyll contents than the WT plants. Moreover, the transcript levels of stress-related genes, such as *NADP-ME*, *H*^*+*^*-Ppase*, *RD29B* and *KIN1* were increased with greater extent in the transgenic plants than the wild plants. On the contrary, *Gshdz4* overexpression lines were much sensitive to osmotic stress at seed germination and stocking stages compared to the wild plants.

**Conclusions:**

We revealed that the important and special roles of *Gshdz4* in enhancing bicarbonate tolerance and responding to osmotic stress. It is the first time to elucidate these novel functions of HD-ZIP transcription factors. All the evidences broaden our understanding of functions of HD-Zip family and provide clues for uncovering the mechanisms of high tolerance of wild soybean to saline-alkaline stresses.

**Electronic supplementary material:**

The online version of this article (doi:10.1186/s12870-016-0872-7) contains supplementary material, which is available to authorized users.

## Background

Adverse environmental factors, such as salt, alkali, and/or drought stresses greatly limit the growth and development of plants which are sessile on the ground. The soil salinity-alkalinity causes crop yield reduction which has become a serious problem for the securities of food supplies [1]. Plants have evolved with complex molecular mechanisms to survive and to keep normal growth under stress conditions. As a result of RNA sequencing profiles of wild soybean roots which were exposed to sodium bicarbonate (NaHCO_3_) [2], a couple of transcription factor genes like *HD-ZIP* [3], *MYB* [4], *WRKY* [5], *NAC* [6], *bZIP* [7], *C2H2* [8] and *TIFY* [9] were identified as alkali stress-responsive genes. Although lots of the previous studies have been reported about the roles of transcription factors in plant tolerance to salt or drought stress, little is known regarding their roles in alkali stress. Therefore, a deep comprehending of the essential mechanisms of plant responses to alkali stress is urgently needed and will contribute greatly to cultivating alkaline-tolerant crop varieties by biotechnology.

In our previous RNA sequencing study [9], we identified an HD-Zip gene from the wild soybean transcriptome. This gene is highly homologous with *Gmhdz4* of cultivated soybean (*G. max*) and encodes a putative homeodomain-leucine zipper protein. The HD-Zip family is unique to the plant kingdom [3]. In a previous study, Chen et al. identified 140 HD-Zip family genes from *G. max* genome and this family can be phylogenetically classified into I, II, III and IV subfamilies [10]. HD-Zip proteins have a conserved homeodomain followed by a leucine zipper in most members or a MEKHLA domain only in subgroup IV members [11]. Homeodomain is a kind of DNA binding domain involved in the transcriptional regulation of key eukaryotic developmental processes and it may bind to DNA as monomers or as homo- and/or heterodimers in a sequence-specific manner. Leucine zipper is consist of respective amino acid sequences on an idealized alpha helix revealed by a periodic repetition of leucine residues at every seventh position over a distance covering eight helical turns [12].

The HD-Zip protein family has been found in a wide variety of plant species, and many efforts have been undertaken to evaluate the functions of HD-Zip genes. HD-Zip I proteins mainly participate in responses to abiotic stresses and the regulation of organ growth and developmental process [3]. For example, the expression of *Oshox22* was strongly induced by salt stress, abscisic acid (ABA), and polyethylene glycol treatment (PEG), and weakly by cold stress in rice [13]. *ATHB7* and *ATHB12* were both strongly induced by abscisic acid (ABA) and water-deficit, and functioned as negative primary regulators of ABA response in *Arabidopsis* [14]. *Zmhdz10*, positively regulated drought and salt tolerance in both rice and *Arabidopsis* [15]. *HB1* from HD-Zip subfamily I of *Medicago truncatula*, was strongly induced by salt stress in root apices and played as regulator of root architecture and lateral root emergence [16]. However, there is little information about HD-Zip I genes responding to alkaline stress.

Bicarbonate stress, including adverse effects of Na^+^, CO_3_^2−^, HCO_3_^−^ and high pH, also elicits negative impacts on plant growth and development. Excessive ion of organic acids such as malate, succinate and citrate were accumulated under bicarbonate (NaHCO_3_) stress, leading to inhibit growth of new root and shoot [17]. The oxidation/reduction-related genes also participate actively in reacting to environmental stimulation which always reflect on physiological indexes such as MDA content and POD activity. Chlorophyll content is also a standard to assess damage of basic cell structure in green plants under stress treatment.

In this study, we explored the functions of *Gshdz4* under alkaline and osmotic stresses. The data showed that there may be different molecular mechanisms and pathways between alkaline stress and osmotic stress and *Gshdz4* functioned as a regulator of bicarbonate stress, providing new evidences to fully understand the functions of HD-Zip genes.

## Results

### Identification and bioinformatics analysis of *Gshdz4*

In our preceding work [2], we noticed that one wild soybean gene which is highly homologous with *Gmhdz4* gene of cultivated soybean was induced by alkaline stress. We designed this gene as *Gshdz4*. Evolution analysis implied that *Gshdz4* belongs to a conserved δ subgroup of HD-Zip I subfamily [10, 18]. Since *Gshdz4* was more significantly up-regulated than the other HD-Zip I members in transcriptome profiling analysis of *Glycine soja* roots treated by alkali stress [2], it was chosen as a further research object in term of functional analyses in this study.

**Fig. 1:**
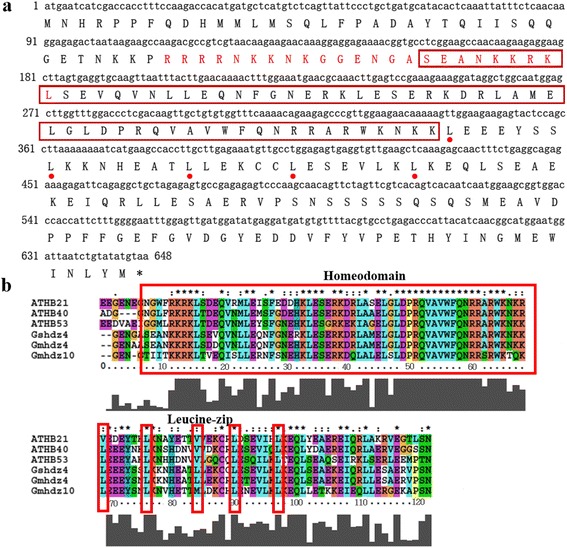
Sequence analysis of *Gshdz4*. **a** *Gshdz4* nucleotide and its deduced amino acid sequences. The homeodomain (HD) and leucine-zip (ZIP) motifs are indicated by *red frame* and *red points*, respectively. One putative nuclear localization signal (NLS) sequence is marked in red. **b** Sequence alignment of HD domains of soybean and *Arabidopsis* subgroup δ members. The conserved leucine residues are indicated by *red frames*

The full-length cDNA of *Gshdz4* was isolated from *Glycine soja* by using homologous cloning strategy. Sequence analysis confirmed that *Gshdz4* contains an open reading frame (ORF) of 648-bp that encodes a protein of 215 amino acids with an estimated molecular weight of 25118.2 Da and a theoretical pI of 6.48. *Gshdz4* protein has a conserved homeodomain (aa57-113) and leucine-zipper domains (aa132-182) (http://www.ncbi.nlm.nih.gov/Structure/cdd/wrpsb.cgi) (Fig. 1b). The cDNA sequence of *Gshdz4* shares high identity to several gene CDS sequences downloaded from the genome database of *Glycine max* in Phytozome (http://phytozome.jgi.doe.gov/pz/portal.html), the green plants genomic database.

A BLASTP search at NCBI showed that Gshdz4 shared 66 % sequence identity with *Glycine max* Gmhdz10 (Glyma02g06560) and 64.7 %, 58.6 %, 66.5 % with *Arabidopsis thaliana* ATHB21 (AT2G18550), ATHB40 (AT4G36740), ATHB53 (AT5G66700), respectively. One putative NLS (nuclear localization signal) motif was predicted to be located in the N-terminus by an online software Predict Protein (https://www.predictprotein.org/) (Fig. 1a).

### Spatial and temporal expression patterns of *Gshdz4*

**Fig. 2:**
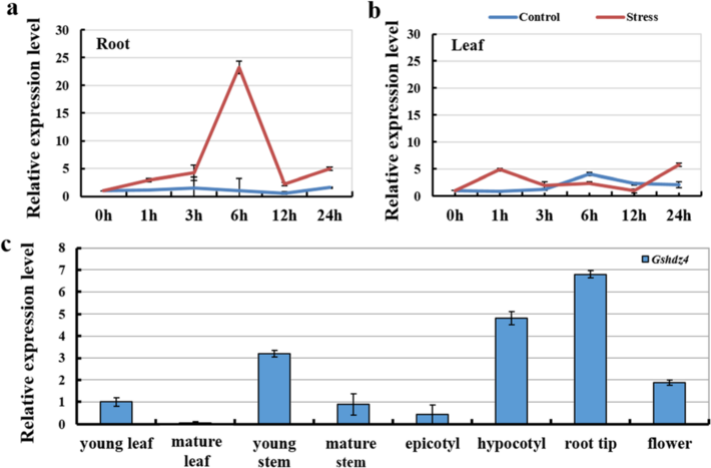
Expression and functional analysis of *Gshdz4*. **a** and **b** *Gshdz4* expression analysis (RT-qPCR) of roots and leaves of 3-week wild soybean seedlings which were subjected to 50 mM NaHCO_3_ treatment. **c** Expression patterns of *Gshdz4* in various tissues of wild soybean. Values represent means of three biological replicates; error bars indicate SD

In order to investigate the induced expression patterns of *Gshdz4* in the roots of wild soybean under 50 mM NaHCO_3_ stress, we carried out qPCR (quantitative real-time PCR) analyses. Under normal condition, the expression of *Gshdz4* kept on a relatively stable level during the whole day and no peak was found (Fig. 2a). However, its transcript level in roots was significantly up-regulated by alkaline stress in a relatively early period of 6 h after treatment (Fig. 2a).

Nevertheless, the expression of *Gshdz4* in leaves was also induced by alkaline stress and reached a peak at 1 h, but reduced to valley at 12 h after treatment (Fig. 2b). It is remarkable that the induced expression level of *Gshdz4* in roots was dramatically higher than that in leaves. The possible reasons for the strong response in roots might be that the plant roots are the exact region of stress perception and the organ directly exposed to stress damage, or the responsive mechanisms for *Gshdz4* expression in roots may differ to that in leaves [19]. Whatever, these results suggest that *Gshdz4* is an alkaline-inducible gene and plays important roles in response toNaHCO_3_ stress in wild soybean.

In addition, the spatial expression of *Gshdz4* was also verified in eight main tissues using qPCR. *Gshdz4* was differentially expressed in all of the tested wild soybean tissues and organs, including young and mature leaves, young and mature stems, epicotyls, hypocotyls, root tips and flowers (Fig. 2c). The results indicated that, among the eight tissues examined in this study, *Gshdz4* exhibited the highest expression level in roots, consistent with its important role of stress resistance in roots. On the contrary, the *Gshdz4* transcript level in leaves especially in old ones was relatively lower, implicating that the diverse mechanisms and roles of *Gshdz4* in roots and leaves [19].

### *Gshdz4* is a nucleus-localized protein

**Fig. 3:**
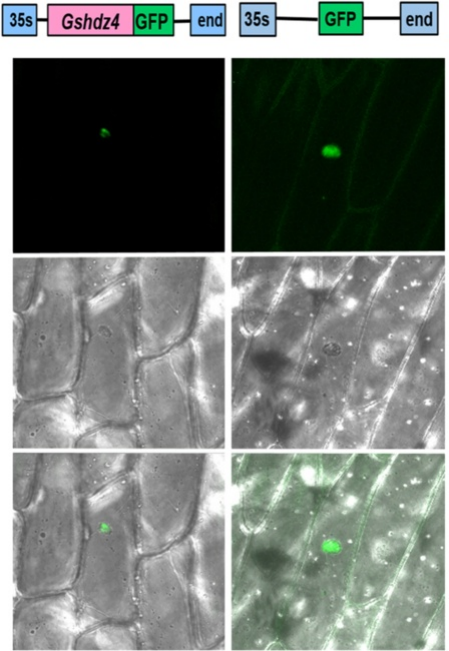
Nuclear localization of *Gshdz4*. Gshdz4-eGFP fusion protein was localized in the nucleus (35S-Gshdz4*-*GFP) and the control was found throughout the cell (35S-GFP)

Sequence analysis showed that *Gshdz4* has one putative NLS motif at the N-terminus and illustrated that *Gshdz4* may target to the nucleus. To affirm the prediction, the pBSK-Gshdz4-eGFP construct was used to investigate the subcellular localization of *Gshdz4* protein in onion epidermal cells through biolistic bombardment. The figures showed that the GFP signal was detected in the nuclei of the onion cells by pBSK-Gshdz4-eGFP construct (Fig. 3). As the control, the eGFP signal produced by pBSK-eGFP construct was found throughout the whole cells (Fig. 3). These results suggest that Gshdz4 should be a nucleus-localized protein which is one of the basic characteristics of transcription factors.

### *Gshdz4* lacks transcription activation activity in yeast cells

**Fig. 4:**
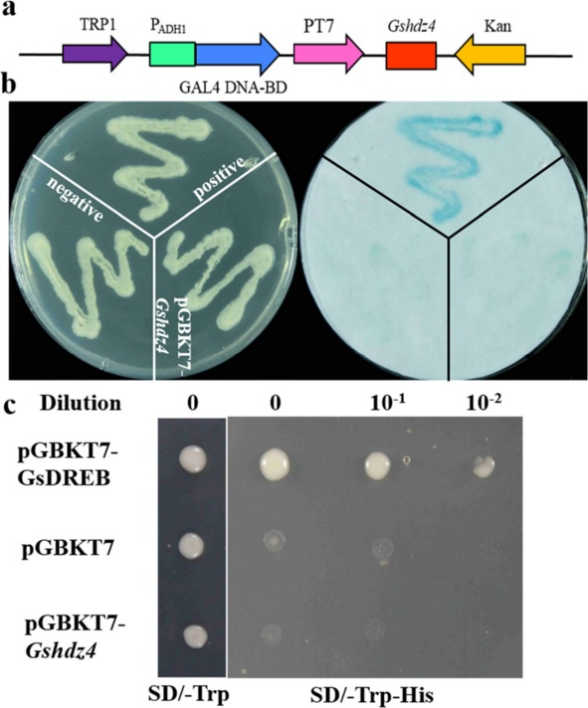
Transcription activity analysis of *Gshdz4*. **a** The construct of pGBKT7-Gshdz4. **b** Galactosidase (LacZ) assay. **c** *His3* reporter assay

To exam whether *Gshdz4* performs trans-activation activity, *Gshdz4* were fused to a DNA binding domain (GAL4), and its ability to activate transcription of *LacZ* and *HIS3* reporter genes was determined. The AH109 yeast strain was transformed with either pGBKT7-GsDREB (positive control), or pGBKT7 (negative control), pGBKT7-Gshdz4,. The AH109 yeast cells carrying one of the above plasmids grew equally well on SD/-Trp medium containing X-gal. However, the positive control performed high galactosidase activity (blue colonies), nevertheless the cells transformed with pGBKT7-Gshdz4, or the negative control, exhibited no galactosidase activity (white colonies) (Fig. 4b). At the same time, the yeast cells carrying the positive control plasmid pGBKT7-GsDREB grew well on SD/-Trp-His medium but the cells with pGBKT7 or pGBKT7-Gshdz4 did not, indicating that *Gshdz4* could not activate the reporter genes expression in yeast cells. It suggested *Gshdz4* may be a transcription repressor or co-activator requires the other plant transcription components.

### Overexpression of *Gshdz4* enhanced tolerance to HCO_3_^−^, but not to OH^−^

**Fig. 5:**
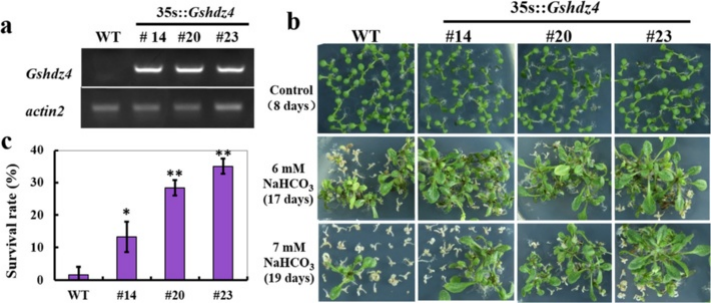
Overexpression of *Gshdz4* enhanced tolerance to NaHCO_3_ stress in germination stage. **a** Transcription levels of *Gshdz4* in transgenic *Arabidopsis* (#14, #20 and #23) and WT by RT-PCR. **b** NaHCO_3_ stress tolerance of *Gshdz4* transgenic *Arabidopsis* (#14, #20 and #23). Plant growth under normal condition and under NaHCO_3_ stress (0, 6 or 7 mM NaHCO_3_). **c** Plant survival rates under NaHCO_3_ stress (0 or 6 mM NaHCO_3_). Values represent means of three biological replicates; error bars indicate SD. Significant differences are denoted with one or two stars if *P* < 0.05 or *P* < 0.01, by Student’s *t*-test

In order to assess the function of *Gshdz4* in alkali stress, *Gshdz4* overexpression lines of *Arabidopsis* were generated. As a result, the *Gshdz4* transgenic plants had similar germination rates with the wild type, although their germination rates showed a little bit of fluctuation in the first 3 days after imbibition (Additional file 1: Figure S1). There was no difference found in growth between the overexpression lines and WT plants in the normal condition (Additional file 2: Figure S3). And then, we compared the germinations and growths of transgenic lines with WT on 1/2 MS medium supplemented with 6 mM or 7 mM NaHCO_3_. Under NaHCO_3_ treatment, the seeds from both *Gshdz4* overexpression lines and WT were capable of developing healthy cotyledons following seed coat breakage and radicle emergence (Fig. 5b) and the germination rate data showed no difference (Additional file 1: Figure S1). However, after 8 days on the medium containing 6 mM or 7 mM NaHCO_3_, the survival rates of transgenic lines were significantly higher than those of WT. The WT plants showed sensitivity to NaHCO_3_ treatments. In addition, on the 20th day after germination, the transgenic lines possessed much higher average percentage of seedlings with open and green leaves (> four green leaves) than WT, and most seedlings of WT turned white and gradually died (Fig. 5b). However, among the three transgenic lines (#14, #20 and #23), #23 line demonstrated the higher survival rate and percentage of green leaves than #20 and #14. These results indicate that overexpression of *Gshdz4* may reduce the damage of high alkaline stress on chlorophyll degradation and may enhance tolerance to NaHCO_3_ to maintain plant growth and development.

**Fig. 6:**
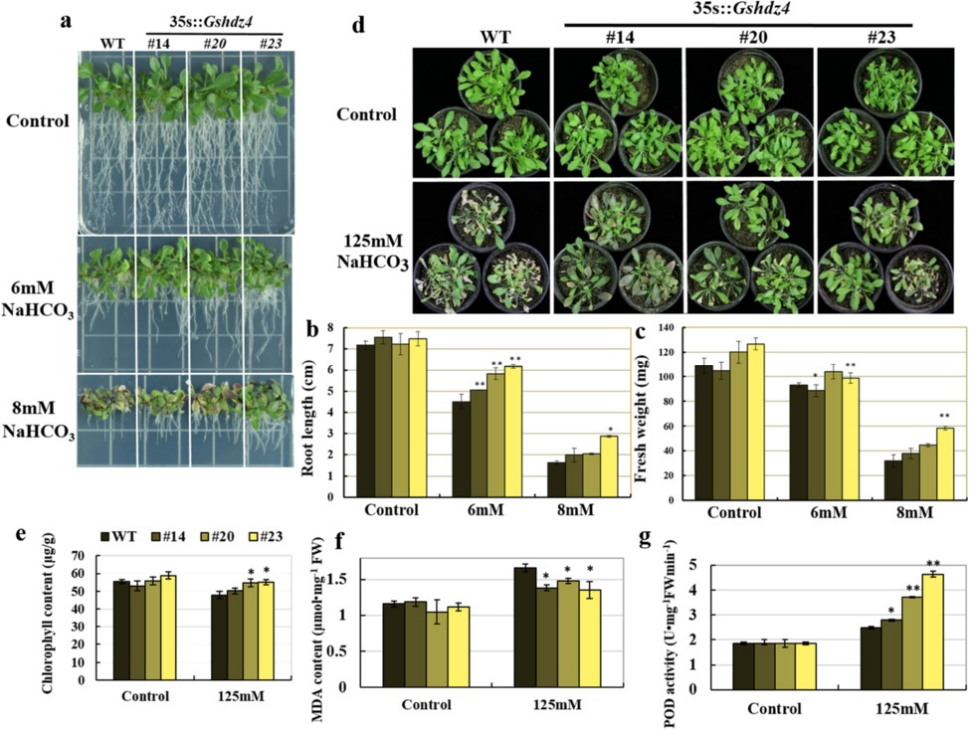
Overexpression of *Gshdz4* enhanced tolerance to NaHCO_3_ stress in seedling and stocking stages of transgenic *Arabidopsis*. **a** *Gshdz4* transgenic *Arabidopsis* in the seedling stage under 0, 6 or 8 mM NaHCO_3_ stress. **b** and **c** Root length and fresh weight of WT and transgenic lines under 0, 6 or 8 mM NaHCO_3_ treatments. **d** *Gshdz4* transgenic *Arabidopsis* (#14, #20 and #23) in the stocking stage under 0 or 125 mM NaHCO_3_ stress. **e**, **g** and **f** Physiological indices of *Gshdz4* transgenic *Arabidopsis* under NaHCO_3_ treatments. Values represent means of three biological replicates; error bars indicate SD. Significant differences are denoted with one or two stars if *P* < 0.05 or *P* < 0.01, by Student’s *t*-test

*Gshdz4* OX also enhanced plant tolerance to NaHCO_3_ stress at the seedling stage. 7 days after germinating, the seedlings of *Gshdz4* OX and WT were transferred onto 1/2 MS medium supplemented with 0 mM, 6 mM or 8 mM NaHCO_3_. The result showed that the growth and development of the WT plants were severely inhibited compared with those of *Gshdz4* OX plants under NaHCO_3_ treatments (Fig. 6a). *Gshdz4* OX lines had longer primary roots and more total weight than WT under alkaline stress (Fig. 6b, c), implying that *Gshdz4* may play an important role in root development and water retention to enhance plants tolerance to NaHCO_3_ stress.

We further investigated the tolerance of *Gshdz4* OX lines and WT to NaHCO_3_ stress at stocking stage. The 3-week-old soil-grown plants were irrigated with 125 mM NaHCO_3_ every 3 days for 2 weeks. The survey data showed that the transgenic plants demonstrated better stress tolerance to NaHCO_3_ than the WT (Fig. 6d). Under normal condition, almost no difference was found in plant growth, the contents of total chlorophyll and malondialdehyde (MDA), and POD activity of the WT and three transgenic lines. However, in the present of 125 mM NaHCO_3_, the total chlorophyll contents decreased in WT more than transgenic plants (Fig. 6e). These results give evidence of that *Gshdz4* OX reduces the damage of high alkaline stress on plants and alleviated chlorophyll degradation. As an indicator of oxidative damage, the content of MDA generated during peroxidation of membrane lipids is often used under abiotic stresses [20, 21]. Thus, the MDA contents were measured in the transgenic and WT plants under alkaline stress conditions and found that the WT accumulated obviously higher levels of MDA than *Gshdz4* OX lines (Fig. 6f). Since the formation of reactive oxygen species (ROS) in the cells can be promoted by most abiotic stresses and subsequently hurts the plants [22, 23], we postulate that overexpression of *Gshdz4* can probably inhibit the production of ROS and/or can even clean the oxidative products to protect plants from membrane damage. To confirm this observation, we determined the endogenous POD activity. The data showed that POD activity of each *Gshdz4* OX line was higher than that of WT (Fig. 6g), meaning that transgenic plants have more ability to get rid of ROS products and extra free radicals to protect cell structure from damage.

**Fig. 7:**
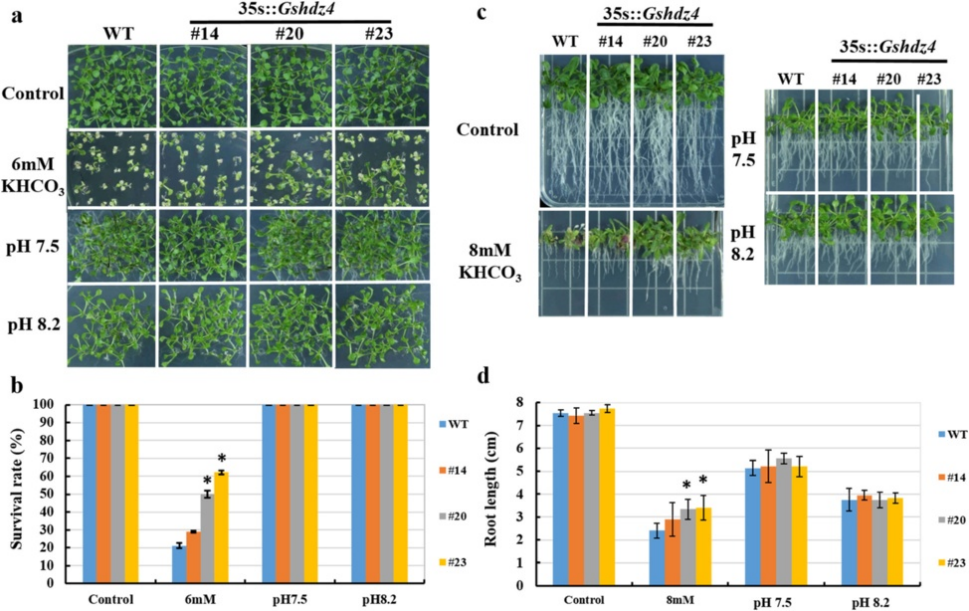
*Gshdz4* transgenic *Arabidopsis* (#14, #20 and #23) under KHCO_3_ (0 or 6 mM) and KOH (pH7.5 or pH8.2) stresses. **a** *Gshdz4* transgenic *Arabidopsis* (#14, #20 and #23) in the germination stage under KHCO_3_ (0 or 6 mM) and KOH (pH7.5 or pH8.2) stresses. **b** Survival rates under KHCO_3_ (0 or 6 mM). **c** *Gshdz4* transgenic *Arabidopsis* (#14, #20 and #23) in the seedling stage under KHCO_3_ (0 or 6 mM) and KOH (pH7.5 or pH8.2) stresses. **d** Root length of WT and transgenic lines (#14, #20 and #23) under KHCO_3_ (0 or 6 mM) stresses. Values represent means of three biological replicates; error bars indicate SD. Significant differences are denoted with one if *P* < 0.05, by Student’s *t*-test

In the alkaline soil, it is HCO_3_^−^ and/or OH^−^-rich environment. For the sake of further investigating if HCO_3_^−^ or OH^−^ or both can generate stress to the plants, the *Gshdz4* OX and WT plants were grown on the medium with 6 mM KHCO_3_ or the medium with pH7.5 and pH8.2 adjusted by KOH. The results showed that all transgenic and WT plants had normal and similar growth on the medium with pH7.5 and pH8.2 adjusted by KOH. However, on the medium supplemented with 6 mM KHCO_3_, the growths of both WT and transgenic lines were greatly inhibited, whereas the transgenic plants had a little higher survival rates than the WT (Fig. 7a). Furthermore, we observed that *Gshdz4* OX and WT seeds had similar germination rates on 1/2 MS medium or 1/2 MS medium supplemented with 6 mM KHCO_3_ or KOH which was used to adjust the medium to pH 7.5 and 8.2, although the germination were delayed to some extent on the medium with 6 mM KHCO_3_ and the medium with pH8.2 (Additional file 3: Figure S2). To further investigate the effect of HCO_3_^−^ or OH^−^ on plant survival and root growth, we grew the 7-day seedlings for 10 days onto the medium containing 8 mM KHCO_3_ or the medium with pH5.8, pH7.5 or pH8.2 adjusted by KOH. The data indicated that the OX and WT plants had similar survival rate and root growth on normal 1/2 MS medium with pH5.8, pH7.5 or pH8.2 but the OX plants had higher survival rate and much better root growth than the WT on the medium containing 6 or 8 mM KHCO_3_ (Fig. 7b, d), implicating that *Gshdz4* overexpression can promote plant tolerance to HCO_3_^−^, but not OH^−^.

### *Gshdz4* regulated the expression of the stress-relative genes under NaHCO_3_ stress

**Fig. 8:**
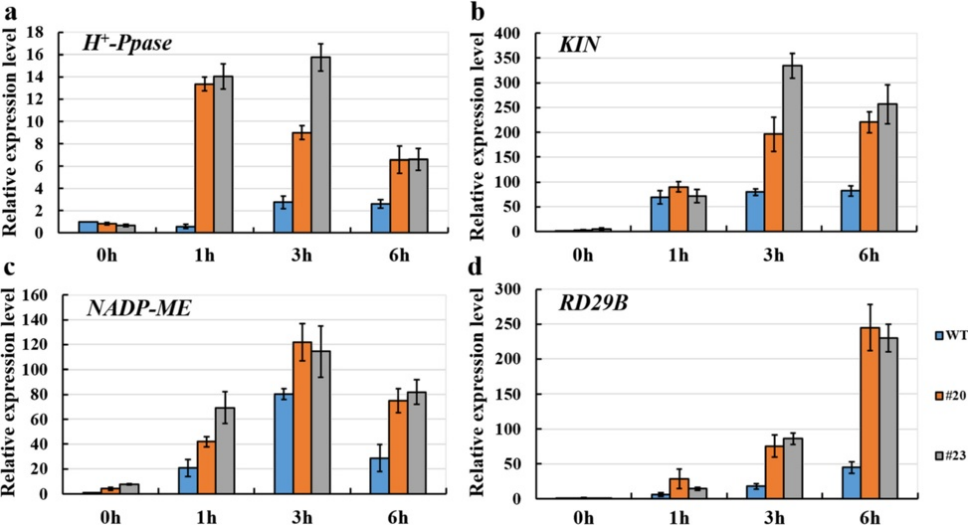
Relative expression levels of alkaline stress-responsive genes in transgenic *Arabidopsis* plants (#20 and #23) under 50 mM NaHCO_3_ stress. **a**, **b**, **c** and **d** showed the expression of *H* ^*+*^ *-Ppase*, *KIN*, *NADP-ME*, *RD29B* respectively. Values represent means of three biological replicates; error bars indicate SD

The alkaline-resistant phenotypes of the *Gshdz4* OX plants indicate that the expression of the stress response genes might be changed in the *Gshdz4* OX lines. To prove this possibility, we compared the expression levels of the representative stress-inducible genes (*NADP-ME*, *H*^*+*^*-Ppase*, *RD29B* and *KIN1*) in WT and *Gshdz4* OX plants under abiotic stress. In the presence of NaHCO_3_ stress, these marker genes demonstrated much higher expression levels in the transgenic plants than the WT plants (Fig. 8a, b, c and d), confirming that *Gshdz4* positively regulates the resistance to NaHCO_3_ stress in plants.

### Overexpression of *Gshdz4* decreased tolerance to osmotic stress in transgenic *Arabidopsis*

**Fig. 9:**
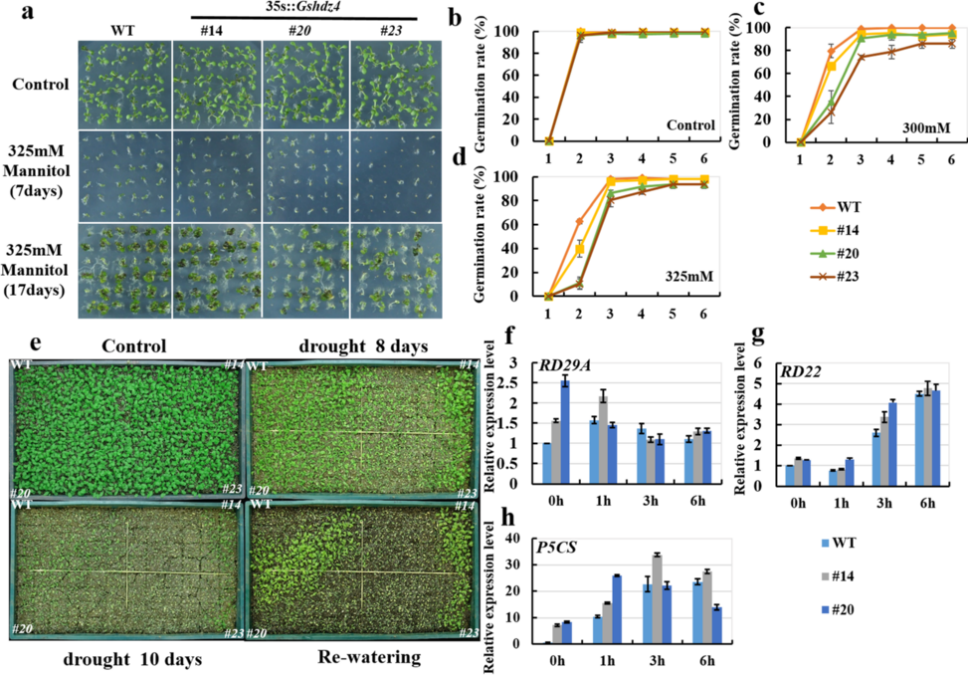
Overexpression of *Gshdz4* enhanced sensitivity to osmotic stress. **a** *Gshdz4* transgenic *Arabidopsis* (#14, #20 and #23) in the germination stage under osmotic stress (0 or 325 mM mannitol) for 7 and 17 days. **b**, **c** and **d** Germination rates under osmotic stress (0 or 325 mM mannitol). **e** *Gshdz4* transgenic *Arabidopsis* (#14, #20 and #23) in the stocking stage under drought stress. **f**, **g** and **h** Relative expression levels of osmotic stress-responsive genes in transgenic *Arabidopsis* plants (#14 and #20)

Since there is some evidence for cross-talk between signaling pathways which regulate different responses to alkaline and drought stresses [24, 25], we investigated and compared the drought-tolerant phenotypes of the *Gshdz4* OX lines and WT plants. Under 325 mM mannitol stress, the seeds of *Gshdz4* OX lines had a slower pace of seed coat breakage and radical emergence than those of WT (Fig. 9a, b, c, d). In additionally, the seeds of *Gshdz4* overexpression lines were unable to develop healthy cotyledons after breakage of seed coats, especially #20 and #23 (Fig. 9a). The overexpression lines exhibited less open leaves as well as green leaves than the WT at the seedling stage (Fig. 9a). At the adult stage of the WT and transgenic plants, all plants were stopped watering for 10 days to make the soil dry entirely. As shown in Fig. 9e, compared to the WT plants, *Gshdz4* OX plants became severely wilted and impaired after dehydration stress. Together, the data indicate that overexpression of *Gshdz4* enhanced drought sensitivity.

To further confirm our observation that *Gshdz4* plays a negative role in plant drought-resistant signaling pathway, we compared the relative expression of osmotic stress response genes (*RD29A*, *RD22*and *P5CS*) in the WT and *Gshdz4* OX plants. The result showed that there was no significant difference between WT and transgenic lines in relative expression levels of these genes, suggesting that *Gshdz4* may negatively regulate plant drought-resistance through the other pathways.

## Discussion

The saline and alkaline stresses are the main environmental stimulation limiting crop growth and leading to crop productivity reduction around the world [26]. Thus, understanding the mechanisms of the plant responses to alkaline or bicarbonate stress, and excavating bicarbonate-resistant genes, will promote biotechnological efforts to cultivate crop plants with enhanced resistant to bicarbonate-rich conditions [9]. Due to its strong resistance to alkaline and salt stresses, wild soybean is an ideal model for the research of the molecular mechanisms of bicarbonate and salt tolerances. A couple of transcription factors were found to be inducible in the transcriptome profiles of wild soybean roots which were treated with alkaline stress specifically. Among these transcription factors, *Gshdz4* is one of highly inducible genes by alkaline stress [2]. They were proved to be diversely expressed in the process of alkaline stress response based on transcriptome profile analysis.

*Gshdz4* belongs to HD-Zip protein family. The name is assigned based on its high homology with the sequences of *G. max* reported in previous study [10]. HD-ZIP family has four subgroups (I, II, III and IV). In *Arabidopsis*, HD-ZIP I genes play a part in response to abiotic stresses, ABA, blue-light and de-etiolation [3]. HD-ZIP II genes respond to illumination conditions [27], shade avoidance [28–30] and auxins [31, 32]. Moreover, several HD-ZIP I subfamily members, like *ATHB7* and *ATHB12* [14], *Hahb-4* [33], *MtHB1* [16], *Oshox22* [13] and *Zmhdz10* [15, 34], were involved chiefly in plant responses to abiotic stresses. Whereas, no any HD-Zip I proteins from *Glycine soja* have been verified and their physiological and biological functions have still unknown until now. In our study, a novel HD-Zip I protein from subgroup δ, *Gshdz4*, was first identified from *Glycine soja* and then was functionally characterized for its response and tolerance to drought and alkaline stress in transgenic *Arabidopsis* plants.

As it was shown in Fig. 1a, the amino acids in red frame were homeodomain and the following leucine residues marked with red points were leucine-zip domain. And the amino acids in red were NLS sequences predicted by the online software. So *Gshdz4* has basic structures similar to homologs from *Glycine max* and *Arabidopsis* and may locate in nucleus as common transcription factors. Sequence analysis affirmed that *Gshdz4* shared high structural homology with *Gmhdz4*, a soybean HD-Zip protein. The results of spatial and temporal expression patterns suggested that *Gshdz4* was significantly induced by NaHCO_3_ stress treatment, espposecially in roots (Fig. 2a) but not in leaves (Fig. 2b). It is possible that the plant roots are the major signal perception organ of soil stresses, which induces *Gshdz4* to express in roots but not in leaves. In analysis of tissue-specific expression patterns, we found that *Gshdz4* had higher levels in young stem, hypocotyl and especially in root tip than the other organs of wild soybean. The transient expression and yeast assays of *Gshdz4* showed that the full-length gene could not activate any reporter genes, giving the possibility that *Gshdz4* may act as a repressor, or a co-activator requiring additional factors to fulfill its function. In cotton, *GhHOX3* is an HD-Zip family gene and is related with cotton fiber elongation and contributes to promoting cell expansion during leaf growth [35, 36]. *GhSLR1* interferes with the *GhHOX3-GhHD1* interaction and represses target gene transcription [35]. In *Arabidopsis*, the transcriptional repression of BODENLOS is controlled by an HD-Zip transcription factor, HB5 [37]. On the other hand, maize HD-Zip, *Zmhdz10*, acts as transcriptional activators [15].

In this study, we first characterized a wild soybean HD-Zip I subfamily gene, *Gshdz4,* which can enhance plant tolerance to HCO_3_^−^, but not OH^−^ (high pH). As shown in the phenotypic data, the transgenic *Arabidopsis* lines (#14, #20, #23) showed significant tolerance to NaHCO_3_ at the germination, young seedling and mature seedling stages. Although there was no difference on germination rates among all lines, the transgenic seedlings possessed higher survival rate, greater root length and heavier fresh weight than the WT, suggesting that overexpression of *Gshdz4* enhanced plant tolerance to alkaline stress. The current knowledge tells us that the alkaline stress in soil is mainly caused by high concentration of NaHCO_3_ [26]. Our data showed that overexpression of *Gshdz4* resulted in enhanced plant tolerance to KHCO_3_, but there was no obvious phenotype difference observed in KOH stress treatment at germination and seedling stages. Previous study suggests that under alkaline stress, plants might may activate much complicated responsive mechanisms in comparison with the plants under other stresses [2]. This may be led by the multiple toxicities of alkali stress, such as high pH, HCO_3_^−^, CO_3_^2−^, and concomitant Na^+^ in NaHCO_3_ solution [38, 39]. As we still don’t know the exact function of *Gshdz4* in regulating alkaline stress, the further investigation is needed to dissect the mechanisms.

Our *Gshdz4* transgenic *Arabidopsis* showed not only phenotypically increased tolerance to HCO_3_^−^ stress, but also transcriptionally increased expression levels of stress-responsive genes, including *NADP-ME*, *KIN1*, *RD29B* and *H*^*+*^*-Ppase*. Most genes modulated by bicarbonate are involved in metabolism, transcription and signaling transduction [40]. In the previous studies, *V-H*^*+*^*-PPase, PEPcase* and *NADP-ME* were reported to be induced by bicarbonate stress [41, 42]. These enzymes play significant roles in intracellular pH regulation, assisting plant cells in dealing with the potential acidification of the cytoplasm under environmental alkaline stress condition. *Gshdz4* may modulate the process of bicarbonate stress tolerance through inducing the high expression of bicarbonate defense genes, such as *NADP-ME* and *H*^*+*^*-Ppase* directly. *KIN1* and *RD29B* are mainly induced by abscisic acid (ABA), cold and drought [43, 44]. The accumulation of these proteins contributes to adjusting physiological conditions in plant cells [45]. The ability of *Gshdz4* to up-regulate *KIN1* and *RD29B* indicates that it may act as an important regulator in the process of bicarbonate resistance and participates in coordinating the expression of plant defense genes. However, growth regulation of plants in response to environmental stresses is extremely flexible and complex, and the complete molecular basis should be investigated further.

In order to defend the oxidative stresses, redox (oxidation-reduction)-related genes also play a key role in environmental stresses resistance, which can scavenge the reactive oxygen and then master the redox balance [2, 23, 46, 47]. At the cellular level, alkaline stress causes the production of active O_2_ species, which result in the peroxidation of membrane lipid. MDA is an end product of lipid peroxidation. Our data showed that lower MDA content and higher POD activity existed in the transgenic plants than in the WT (Fig. 6f, g), implying that the degree of membrane injury of transgenic plants was less than that of WT, consistent with the enhanced alkaline tolerance phenotype of transgenic *Arabidopsis*.

Similarly, the transgenic plants showed greater tolerance to bicarbonate stress than the WT (Fig. 6d). In the absence of NaHCO_3_, there was almost no difference no matter in growth of plants or in contents of total chlorophyll among the WT and three transgenic lines. However, after supplement with 125 mM NaHCO_3_, the total chlorophyll contents in WT plants decreased more than in transgenic plants (Fig. 6e). These results suggested that overexpression of *Gshdz4* reduced chlorophyll degradation and enhanced tolerance to bicarbonate in transgenic plants. However, the exact mechanism of *Gshdz4* in this aspect is still unknown.

To our surprise, the negative regulation of drought stress by *Gshdz4* was observed. Phenotypic experiments at both germination and seedling stages revealed that transgenic plants showed more sensitivity to osmotic stress than the WT, implying that *Gshdz4* regulates alkaline and osmotic stresses by different ways. To better understand the molecular basis of the impaired drought stress tolerance of the *Gshdz4* transgenic plants, the expression patterns of the stress-related genes, such as *P5CS*, *RD22* and *RD29A*, were investigated. These genes have been used as convenient markers for monitoring the ABA and stress-responsive pathways in plants [48]. However, there was no significant difference observed in the experiment, implying that *Gshdz4* may not regulate response to drought stress via these genes.

## Conclusions

We identified a novel HD-ZIP I gene, *Gshdz4*, from *Glycine soja. Gshdz4* encodes a protein with conserved homeodomain-leucine zipper domain and acts as a positive regulator in alkaline (bicarbonate) stress in *Arabidopsis*. The overexpression of *Gshdz4* displays a significant tolerance to NaHCO_3_ stress in the seed germination, seedling growth and stocking stage. Moreover, transgenic lines showing tolerance to KHCO_3_ but not to KOH indicated that *Gshdz4* may play an important role in bicarbonate tolerance pathway. What is more interesting is that the overexpression of *Gshdz4* showed sensitivity to osmotic stress and did not regulate the marker genes in osmotic stress-relative pathways. Our studies broaden our knowledge level about HD-ZIP I family gene functions, and complement the evidences which was developed in previous studies.

## Methods

### Plant materials and growing condition

The seeds of *G. soja* G07256 were gained from Jilin Academy of Agricultural Sciences which is located in Changchun, China. At the first of the process, we treated the seeds with sulfuric acid (98 %) for 10 min, washed them for 5 times using sterile water after which they were kept in darkness with a little sterile water for at least 2 days to facilitate germination. Following that, we transferred the seedlings to make them grow on 1/4 Hoagland solution for 19 days at 24–26 °C and in 16 h light/8 h dark cycles. To study the tissue-specific expression of *Gshdz4*, the samples of young leaves, old leaves, young stems, old stems, epicotyls, hypocotyls, roots and flowers were taken from the wild soybean plants. To detect the expression profile of *Gshdz4* gene under alkali-stress treatment, the 19-day seedlings were kept in 1/4 Hoagland liquid mediums containing 50 mM NaHCO_3_ (pH 8.5) for 0, 1, 3, 6, 12 or 24 h, respectively. All the samples were stored at −80 °C after snap-frozen in liquid nitrogen.

*Arabidopsis thaliana* Columbia (Col-0) was used as the wild-type plant for transformation. The WT and transgenic seeds of *Arabidopsis* were grown in a greenhouse under the controlled environmental conditions (21–23 °C, 100 μmol photons m^−2^ s^−1^, 60 % relative humidity, 16 h light/8 h dark cycles). The samples were collected as mentioned above.

### Identification and bioinformatics analysis of *Gshdz4*

The cDNA (full-length) of *Gshdz4* was obtained by homologous cloning. The total RNA of *G. soja* G07256 was isolated from leaves by EasyPure RNA Kit (Transgen Biotech) and was converted to cDNA by reverse transcription using GoldScript cDNA Synthesis Kit (Invitrogen). The full-length cDNA of *Gshdz4* was amplified using gene-specific primers: 5′-AAGCCAGTGAAGAGCGAGCGAGA-3′ and 5′-CTACCATTTCGGGTCAAATTGCAAA-3′. The PCR product was cloned into the pEASY-Blunt Zero Cloning Vector (Transgen Biotech) and subjected to sequencing. Sequence alignments were performed using DNAMAN. Phylogenetic analysis was carried out with MEGA 4.0. Similarity of sequences was verified by the BLASTP at NCBI online (http://blast.ncbi.nlm.nih.gov/Blast.cgi). Homology searches were done by Phytozome (http://www.phytozome.net/soybean).

### Subcellular localization analysis

*Gshdz4* CDS was amplified from pEASY-Blunt Zero Cloning Vector by PCR with *Bam*HI and *Spe*I linker primers: 5′- TAATATGGATCCCATGAATCATCGACCACCTTTC-3′ and 5′-AATTATACTAGTGCATATACAGATTAATCCATTCCATG-3′. The full-length *Gshdz4* gene was cloned into pBSK-eGFP vector. The recombinant Gshdz4-green fluorescent protein (GFP) plasmid was bombarded into epidermal cells of onion with particle bombardment. Analysis of GFP was performed as mentioned previously [49].

### Transcriptional activation analysis

The full-length CDS (coding sequence) of *Gshdz4* was amplified by PCR using *Nde*I and *Sal*I linker primers: 5′- TAATATCATATGGGATGAATCATCGACCACCTTTC-3′ and 5′- AATTATGTCGACGTTACATATACAGATTAATCCATTCCATG-3′. The gene was introduced into pGBKT7 vector. The pGBKT7-GsDREB (positive control) and the pGBKT7 (negative control) were transformed into yeast strain AH109. Transcription-activation activity was analyzed following the protocols mentioned previously [50].

### Generation of transgenic *Arabidopsis thaliana*

The pCAMBIA330035Su-Gshdz4 plasmid was constructed by amplifying the full-length CDS of *Gshdz4* from pEASY-Blunt Zero Cloning-Gshdz4 by PCR using primers: 5′-GGCTTAAUAAGCCAGTGAAGAGCGAGCGAGA-3′ and 5′-GGTTTAAUCTACCATTTCGGGTCAAATTGCAAA-3′. The underlined nucleotide sequences indicate *Pac*I restriction enzyme cutting site. The gene was cloned into a USER (uracil-specific excision reagent) vector pCAMBIA330035Su as describe previously [51]. The resulted vector was mobilized into *Agrobacterium tumefaciens* strainGV3101. Plant transformation was performed by using the floral dipping method [52]. The transgenic plants from T_1_ generation were screened on 1/2 MS solid mediums containing 25 mg L^−1^glufosinate. The seeds from each T_1_ plant were individually collected. The selected T_2_ plants were sowed, and the transgenic lines were confirmed by RT–PCR assay (Fig. 5a). The homozygous overexpression lines (#14, #20 and #23) were obtained by PCR and the progeny segregation in T_3_ generation.

### Phenotypic analysis of transgenic *Arabidopsis* under the treatments of NaHCO_3_, KHCO_3_, KOH and drought

We selected three T_3_ homozygous overexpression lines (# 14, # 20, and # 23) to study their resistance to various stresses. The WT and homozygous transgenic *Arabidopsis* seeds were sterilized and were sown on 0.5× MS agar medium, which were supplemented with 0, 6, 7 mM NaHCO_3_, or 6 mM KHCO_3_ or 325 mM mannitol or KOH to make the medium at pH 7.5 or pH 8.2. For root length and fresh weight assays, the seeds were germinated on 0.5× MS agar medium for 7 days and then were transferred to fresh medium supplemented with 0, 6, 8 mM NaHCO_3_, 6 mM KHCO_3_, 325 mM mannitol, or KOH to adjust the medium to pH 7.5, pH 8.2. The seedlings were grown on the medium for 12–14 days and the lengths of roots were measured. All experiments were performed 3 completely independent biological replicates at least. All statistical analysis was done with EXCEL2010 (Microsoft, http://www.microsoftstore.com.cn/).

To study of *Gshdz4* transgenic *Arabidopsis* phenotypes, the seeds from WT and transgenic plants were sown in the soil supplemented with appropriate vermiculite and the 3-week-old plants were treated by water containing either 0 or 125 mM NaHCO_3_ every 3 days for 15 days. To detect drought resistance at the adult stages, water was withheld for 10 days from 21-day-old plants.

The data were analyzed by EXCEL 2010 and the results from one representative experiment were shown (all assays were repeated at least three times).

### Quantitative real-time PCR analysis of *Gshdz4* and stress response genes

Total RNA was extracted from *G. soja* or *Arabidopsis* seedlings by using EasyPure RNA kit (Transgen Biotech), and cDNA synthesis was performed using the GoldScript cDNA Synthesis kit (Invitrogen, Carlsbad, CA, USA). cDNA quality was assessed by PCR using specific primers for *GAPDH* (glyceraldehyde-3-phosphate dehydrogenase, accession no. DQ355800) to exclude genomic DNA contamination. The qRT-PCR was performed in 96-well (25 μL) format using the SYBR Premix ExTaq™ II Mix (TaKaRa, Shiga, Japan) on an ABI 7500 sequence detection system (Applied Biosystems, USA). One microliter of each synthesized cDNA (diluted 1:5) was used as template. Amplification of *GAPDH* in *G. soja* or *ACTIN2* in *Arabidopsis* was used as controls. Expression levels for all candidate genes were determined using the 2^–ΔΔCT^ method. The relative transcript levels were calculated and normalized as described previously [53]. cDNA from three independent plants were treated as three biological replicates and each cDNA sample was repeated three times as three technical replicates, and the results from one representative experiment are shown.

### Chlorophyll content, POD activity and MDA content

For measurement of chlorophyll content, 1 g of fresh leaf samples from transgenic lines and WT plants were quickly homogenized in 0.5 mL of 100 % acetone followed by addition of 1 mL of 80 % acetone to determine the chlorophyll contents according to the method described previously [54]. The absorbance values of chlorophyll *a* and chlorophyll *b* were respectively measured at 663 and 645 nm using an ultraviolet spectrophotometer (UV-2550, Shimadzu, Tokyo, Japan).

To assess peroxidase (POD) activity, fresh leaf segments from the same locus of each plant were respectively homogenized in 0.05 mol/L phosphate buffer (pH 5.5). The homogenates were transferred into centrifuge tubes and were centrifuged at 4000 rpm for 10 min. The absorbance at 470 nm was measured using an ultraviolet spectrophotometer (UV-2550, Shimadzu, Tokyo, Japan).

The MDA content was determined using the thiobarbituric acid (TBA) protocol [55, 56]. Equal amount of leaves from transgenic lines and WT plants were frozen by liquid nitrogen and grinded into powdery in 1 mL 5 % trichloroacetic (TCA). The homogenates were centrifuged at 15,000 rpm for 20 min and the supernatants were taken to mix well with 800 μL of 0.5 % TBA. The reaction mixtures were heated at 95 °C for 30 min and were cooled on ice and then were centrifuged at 15,000 rpm for 20 min again. The absorbance at 450, 532, and 600 nm was respectively measured using an ultraviolet spectrophotometer (UV-2550, Shimadzu, Tokyo, Japan).

## Additional files

Additional file 1: Figure S1. Germination rates under NaHCO_3_ (0, 6 or 7 mM) stress. (TIF 568 kb) Additional file 2: Figure S3. No difference in growth between the overexpression lines and WT plants in the normal condition. (TIF 3134 kb) Additional file 3: Figure S2. Germination rates under KHCO_3_ (0 or 6 mM) and KOH (pH7.5 or pH8.2) stresses. (TIF 549 kb)

## Abbreviations

ABA: Abscisic acid
CTRs: Carboxy-terminal regions
*Gshdz4*: *Glycine soja* homeodomain-leucine zipper 4
*H*^*+*^*-Ppase*: H^+^-pyrophosphatase
*KIN1*: Kinase 1
MDA: Malondialdehyde
*NADP-ME*: NADP-dependent malic enzyme
NLS: Nuclear localization signal
NTRs: Amino-terminal regions
P5CS: 1-Pyrroline-5-carboxylate synthetase
OX: Overexpression
POD: Peroxide
RD22: Responsive to dehydration 22
RD29A: Responsive to dehydration 29A
RD29B: Responsive to dehydration 29B
SD: Synthetically defined medium
USER: Uracil-specific excision reagent
WT: Wild type
