# Supplementary figures and images for: A novel Glycine soja homeodomain-leucine zipper (HD-Zip) I gene, Gshdz4, positively regulates bicarbonate tolerance and responds to osmotic stress in Arabidopsis
Source: BMC Plant Biol. 2016 Aug 24;16(1):184. doi: 10.1186/s12870-016-0872-7 (PMC4995822; doi:10.1186/s12870-016-0872-7)

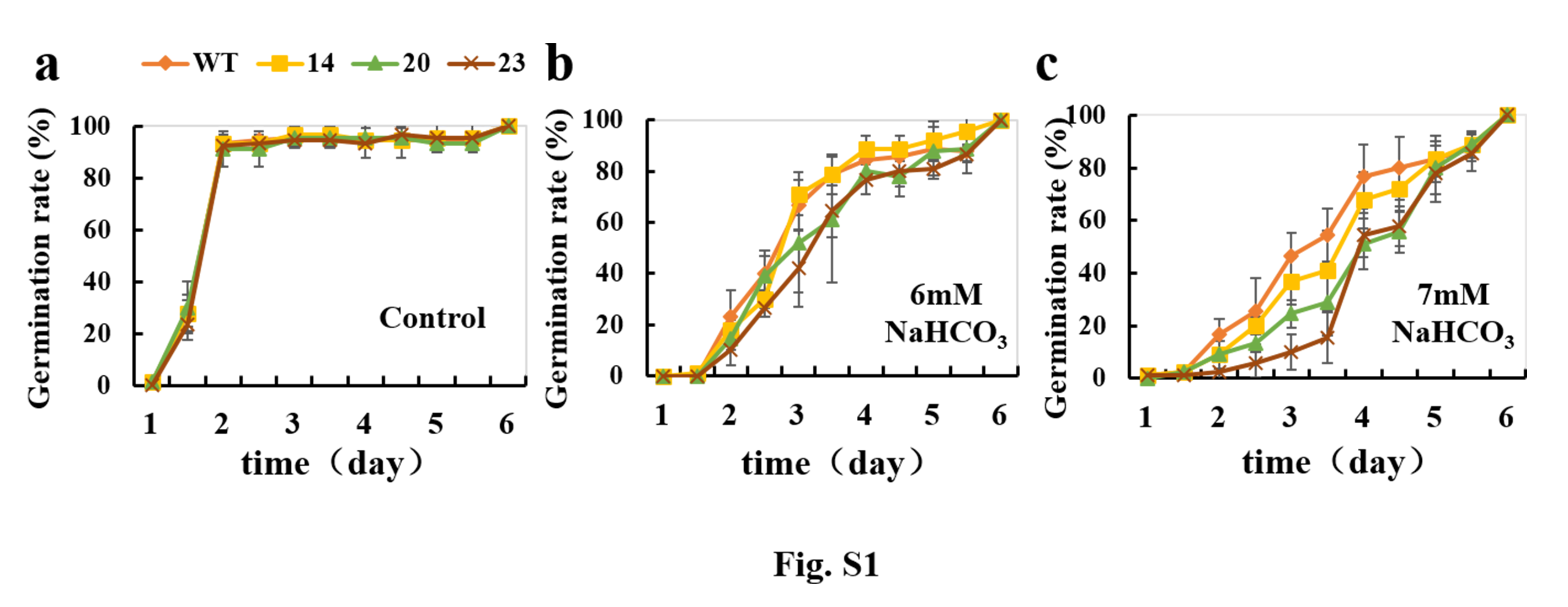

Supplement: Additional file 1: Figure S1. — Germination rates under NaHCO3 (0, 6 or 7 mM) stress. (TIF 568 kb) [file 12870_2016_872_MOESM1_ESM.tif]

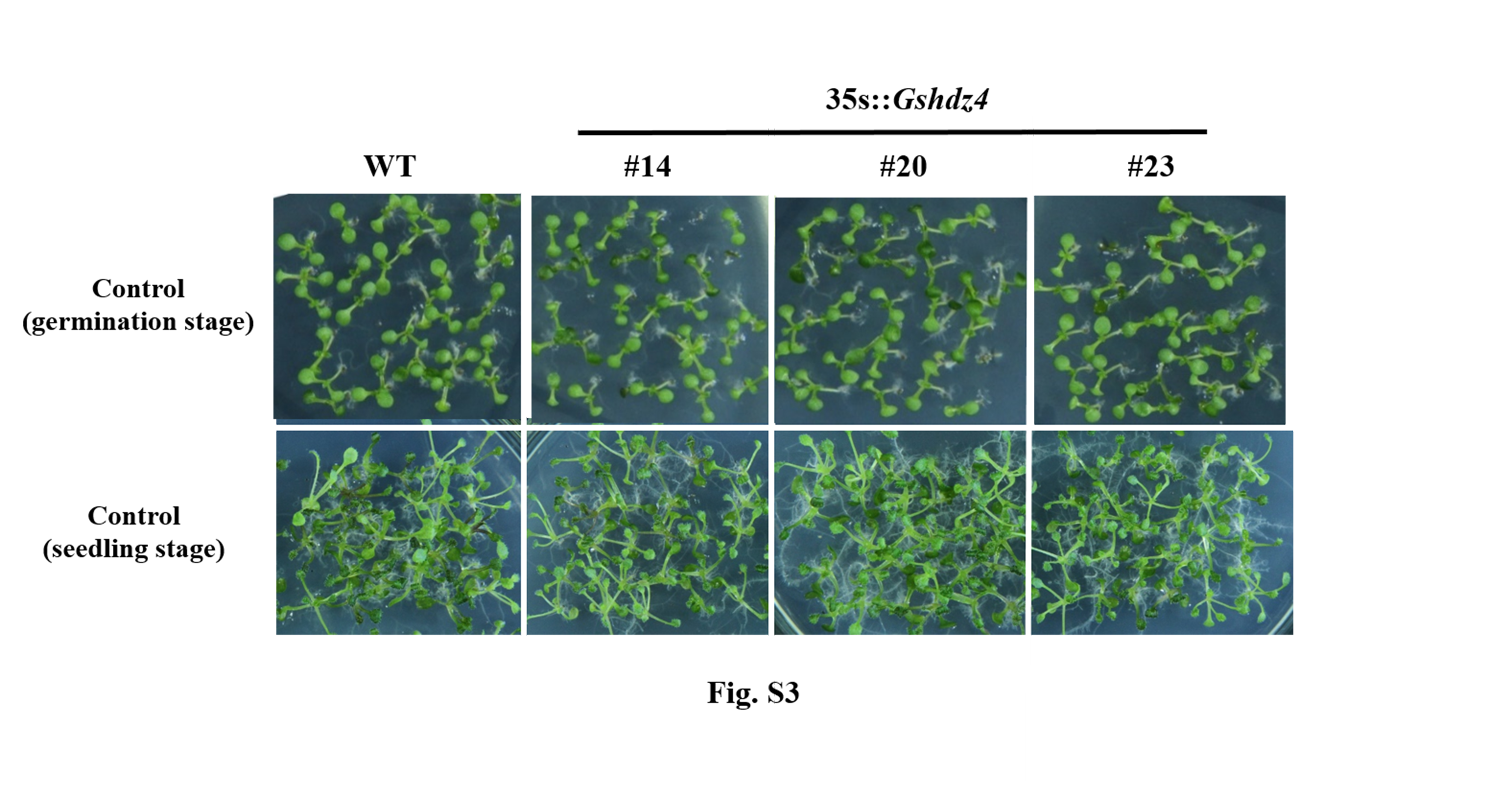

Supplement: Additional file 2: Figure S3. — No difference in growth between the overexpression lines and WT plants in the normal condition. (TIF 3134 kb) [file 12870_2016_872_MOESM2_ESM.tif]

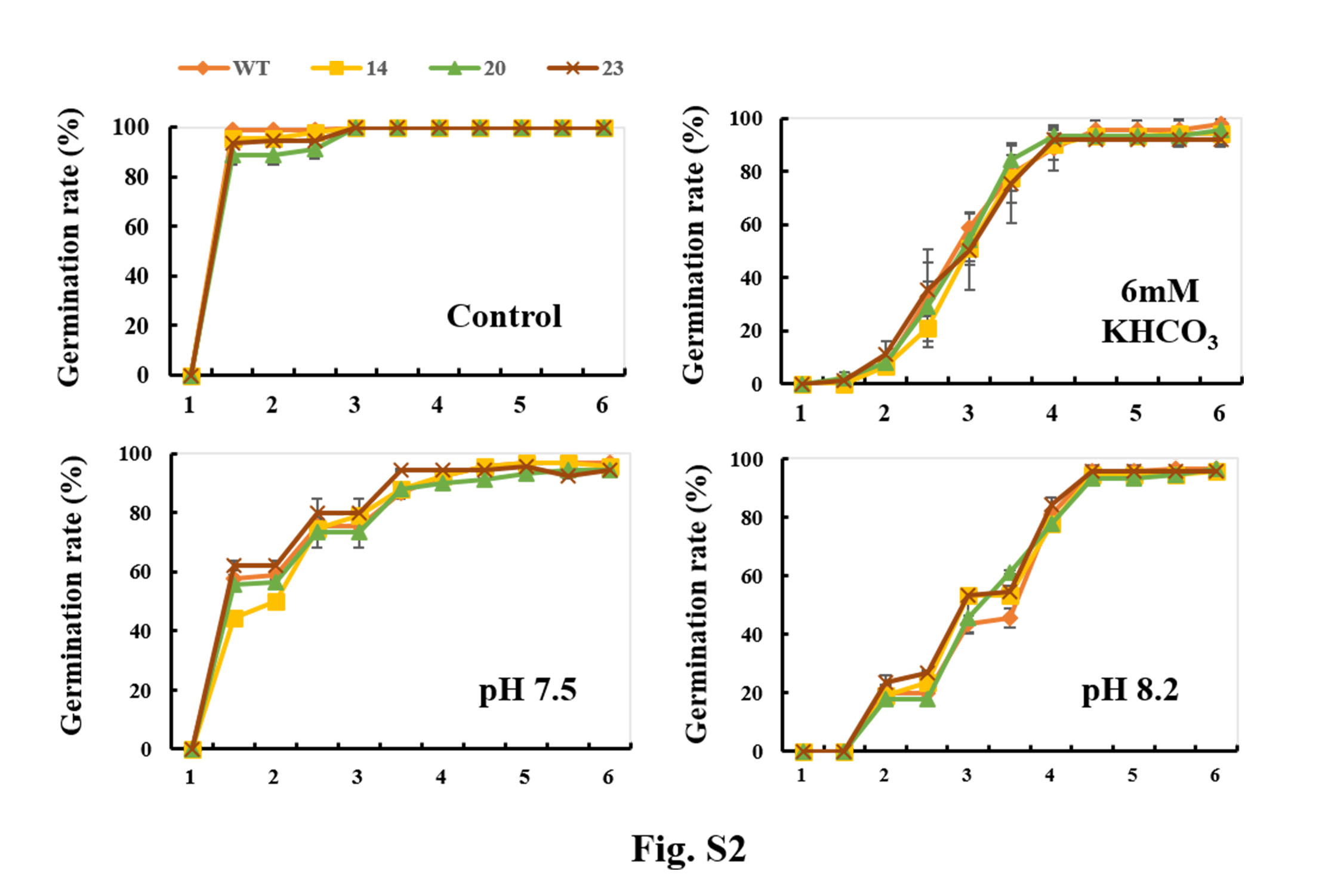

Supplement: Additional file 3: Figure S2. — Germination rates under KHCO3 (0 or 6 mM) and KOH (pH7.5 or pH8.2) stresses. (TIF 549 kb) [file 12870_2016_872_MOESM3_ESM.tif]
